# Does Low-Field MRI Tenography Improve the Detection of Naturally Occurring Manica Flexoria Tears in Horses?

**DOI:** 10.3390/ani15152250

**Published:** 2025-07-31

**Authors:** Anton D. Aßmann, José Suàrez Sànchez-Andrade, David Argüelles, Andrea S. Bischofberger

**Affiliations:** 1 Equine Hospital, Vetsuisse-Faculty, University of Zürich, 8057 Zürich, Switzerland; anton.assmann@uzh.ch; 2 Clinic for Diagnostic Imaging, Vetsuisse-Faculty, University of Zürich, 8057 Zürich, Switzerland; jsuarez@vetclinics.uzh.ch (J.S.S.-A.); abischofberger@vetclinics.uzh.ch (A.S.B.); 3 Equine Veterinary Teaching Hospital, University of Cordoba, 14001 Cordoba, Spain

**Keywords:** MRI, tenoscopy, horse, manica flexoria

## Abstract

Diagnosing digital flexor tendon sheath (DFTS) pathologies, particularly manica flexoria (MF) tears, can be challenging with standard imaging modalities. Standing low-field MRI tenography (MRIt) may improve the detection rate of MF tears. This study aimed to compare ultrasonography, contrast radiography, pre-contrast MRI, and MRIt to detect naturally occurring MF lesions in horses undergoing tenoscopy. Ten horses with a positive DFTS block, which underwent contrast radiography, ultrasonography, MRI, MRIt, and tenoscopy were included. Two radiologists evaluated the images and recorded whether an MF lesion was present and determined the lesion side. Sensitivity and specificity were calculated for each modality using tenoscopy as a reference. MRIt and contrast radiography detected MF lesions with the same frequency, both showing 71% sensitivity and 100% specificity. Pre-contrast MRI and ultrasonography detected MF lesions with a lower sensitivity (57%); however, the MRI (100%) demonstrated a higher specificity than ultrasonography (33%). Adding contrast in MRI changed the sensitivity from (4/7 lesions) 57% to (5/7 lesions) 71%, with a constant high specificity (100%). MRIt diagnoses MF tears with a similar sensitivity to contrast radiography, with the same specificity, but with the added benefit of lesion laterality detection. The combined advantages of the anatomical detail of the T1 sequence and the post-contrast hyperintense appearance of the fluid may help diagnose MF tears and identify intact MFs. However, this needs to be substantiated in a larger number of cases.

## Simple Summary

Diagnosing digital flexor tendon sheath (DFTS) pathologies, particularly manica flexoria (MF) tears, can be challenging with standard imaging modalities. Standing DFTS MRI tenography (MRIt) may improve the pre-operative diagnosis of MF lesions. This study aimed to assess the diagnostic performance of ultrasonography, contrast radiography, native MRI, and MRIt for detecting naturally occurring MF lesions in horses undergoing tenoscopy. Ten horses with a positive DFTS block, ultrasonographic and contrast radiographic examination, MRI, MRIt, and tenoscopy were included. Two radiologists retrospectively evaluated the images and recorded whether an MF lesion was present. Sensitivity and specificity were calculated for each modality using tenoscopy as the reference, and the values were compared using McNemar’s tests. MF lesions were identified with equal frequency using the MRIt and contrast radiography, both showing a sensitivity of 71% and a specificity of 100%. The MRI and ultrasonography detected MF lesions with the same sensitivity of 57.1%, but the MRI demonstrated higher specificity compared to ultrasonography (100% and 33%). There was no statistically significant difference between the imaging modalities for detecting MF lesions (*p* = 1). Specifically, MRIt and contrast radiography each detected five out of seven MF lesions, while MRI and ultrasonography each detected four out of seven lesions. MRIt did not enhance the diagnostic performance of low-field MRI to diagnose naturally occurring MF tears in horses. Furthermore, neither the MRI nor MRIt demonstrated superiority over contrast tenography, except in the diagnosis of lesion laterality. Compared with ultrasonography, which exhibited a rather low specificity, both MRI and MRIt may better distinguish an intact from a torn MF.

## 1. Introduction

Tenosynovitis secondary to laterally situated marginal deep digital flexor tendon tears (DDFT) or manica flexoria (MF) tears is a common digital flexor tendon sheath (DFTS) pathology in horses [1,2,3]. However, diagnosing intrathecal tendinopathy, especially MF tears, can be challenging.

Ultrasonography has been reported to have a low diagnostic accuracy in detecting MF tears [4]. In one study, ultrasonography identified MF lesions with a sensitivity of only 38% [3]. Moreover, ultrasonography detected lesions confirmed by tenoscopy in only up to 49% of cases [3]. Another study reported a sensitivity of 64% and a specificity of 92% for the ultrasonographic diagnosis of MF lesions [5]. MF lesions were detected by contrast radiography with a considerably higher sensitivity (92–96%) and specificity (56–80%) [6,7]. Nevertheless, these low specificities represent a significant potential for error, and in many cases, tenoscopy remains the diagnostic tool of choice for DFTS disorders [3,4,8,9].

The highly detailed 3D cross-sectional images captured with computed tomography (CT) and magnetic resonance imaging (MRI) can often reveal pathological changes that radiography and ultrasonography may miss.

Several studies have evaluated the use of CT and CT tenography (CTT) for diagnosing pathologies of the DFTS, demonstrating superior diagnostic performance compared to standard imaging modalities [10,11,12,13]. In a cadaveric model, CTT achieved a sensitivity of 85% and a specificity of 96% in identifying experimentally induced lesions of the MF [13]. Additionally, three clinical reports have described the use of CTT for diagnosing MF lesions in a small number of horses [10,11,12].

However, in many clinics and institutions, MRI is the only standing cross-sectional modality available. MRI offers significant potential for detecting MF injuries due to its superior soft tissue contrast. In terms of resolution and detail, high-field MRI is undoubtedly the gold standard for diagnosing bone and soft tissue pathology, as several studies have shown [14,15]. In recent years, high-field MRI has been replaced by low-field MRI, which allows for imaging of standing, sedated horses [16]. However, this technique is limited by reduced spatial resolution resulting from thicker slices. Despite this limitation, low-field MRI has been successfully used to visualise a variety of pathologies, particularly those affecting soft tissues in the hoof as well as the metacarpophalangeal and metatarsophalangeal regions [17,18,19,20]. However, only a few authors have described MRI application for the diagnosis of tendon lesions within the DFTS [20,21,22]. The choice of MRI sequences plays a crucial role in determining the contrast between different tissue types. For instance, T2-weighted images, in which fluid appears hyperintense relative to surrounding soft tissues, provide greater contrast than T1-weighted images, where fluid and soft tissues often exhibit similar signal intensities [23,24]. Tendon lesions are typically identified on MRI as T2 hyperintense areas within the normally hypointense tendon structure. Despite the superior contrast of T2-weighted sequences for detecting these pathologies, T1-weighted sequences are generally considered superior in terms of anatomical detail and spatial resolution [25].

Injection of a paramagnetic contrast media into the DFTS and acquiring post-contrast T1 sequences combines the advantages of both T1 and T2 sequences and may improve the diagnostic performance of standing low-field MRI, when diagnosing MF lesions. Gadolinium-enhanced MRI techniques have been researched and applied in human medicine for many years [26,27,28,29,30,31,32,33]. The diagnostic benefits are now being noticed in veterinary medicine, and there are some reports of applications in dogs and horses [34,35,36,37,38,39]. The authors have tested the accuracy of MRI in the detection of artificially placed tendon lesions in the tendon sheath in a cadaver model and tested the application of gadolinium-enhanced MRI tenography to improve the visibility of intrathecal tendon lesions [39].

The purpose of this study was to assess the diagnostic performance of ultrasonography, contrast radiography, native MRI, and MRI tenography (MRIt) for detecting naturally occurring MF lesions in horses undergoing tenoscopy and to compare standing low-field MRI and MRIt with these standard diagnostic tools. It was hypothesised that MRIt would best diagnose MF lesions compared to the other imaging modalities.

## 2. Materials and Methods

This was a retrospective, cross-sectional, and method-comparing study. Horses were selected for inclusion in the study if lameness was blocked by intrathecal anaesthesia of the DFTS; the horses underwent standing low-field MRI, MRIt, and tenoscopy. Clinical and diagnostic examinations were performed from 1 March 2021 to 31 August 2023. Lameness examinations were performed by ECVS board-certified equine surgeons at the Equine Hospital, Vetsuisse-Faculty, University of Zürich. Diagnostic anaesthesia of the DFTS was performed by injection of mepivacaine (Mepivacaine Sintetica 20 mg/mL, SINTETICA S.A., 6850 Mendrisio, Switzerland) into the distal pouch of the DFTS. Following blocking, each horse underwent ultrasonography and contrast radiography. Subsequently, all horses underwent standing low-field MRI without and with contrast. Clients of horses participating in the study signed consent forms. After completion of the imaging procedures, all horses underwent diagnostic–therapeutic tenoscopy.

### 2.1. Ultrasonography

Experienced ECVDI board-certified large animal radiologists conducted the ultrasonographic examinations using a LOGIQ S8 Vet ultrasound machine (LOGIQ S8 Vet, GE Healthcare AG, Opfikon, Switzerland) with linear probes ML6–15 (6–15 MHz) and 9 L (2.5–8 MHz). All radiologists followed identical protocols for weight-bearing and non-weight-bearing assessments. The procedure started with a static weight-bearing scan of the palmar/plantar fetlock region, evaluating the DFTS in both longitudinal and transverse planes. Next, a static scan of the flexed, non-weight-bearing limb was performed. Finally, dynamic imaging was performed by having another person lift the limb while the joint was flexed and extended to visualise the tendons and MF. Probe settings, including frequency, depth, and gain, were modified by the operator to enhance image quality.

### 2.2. Contrast Radiography

Contrast radiography of the DFTS was carried out either simultaneously with intrathecal anaesthesia or the next day. The tendon sheath was injected with a mixture of 13 mL of mepivacaine and 7 mL of iodinated contrast agent (Visipaque Iodixanol 270 mg/mL, GE Healthcare AG, Opfikon, Switzerland). To ensure an even distribution of the contrast agent within the sheath, the horse was walked a few steps before a lateromedial radiograph of the DFTS was taken.

### 2.3. Magnetic Resonance Imaging and Magnetic Resonance Imaging Tenography

For standing MRI, a venous catheter was placed into the jugular. All horses were premedicated according to the same protocol: 0.03 mg/kg acepromazine and 0.1 mg/kg morphine intramuscularly 30 min before MRI. Immediately before the examination began, each horse received a bolus of 0.01 µg/kg detomidine intravenously. Sedation was then maintained using detomidine continuous rate infusion. The affected limb was then placed in a 0.27 T MRI magnet (Hallmarq Veterinary Imaging, Ltd., Surrey, UK) with the Fetlock RF Coil (Hallmarq Advanced Veterinary Imaging, Hallmarq Veterinary Imaging Ltd., Guildford, Surrey, UK) applied. A short protocol was used: pre-contrast, a T2 FSE MI transverse, 5 mm slice thickness and a T1 3D GRE transverse, 5 mm slice thickness of the proximal recess of the DFTS were obtained. Afterwards, 0.5 mL of the gadolinium contrast agent (0.5 mmol/mL) (Dotarem, Gadotersäure, Guerbet AG, Zürich, Switzerland) diluted in 20 mL 0.9% NaCl was injected into the DFTS in a standard aseptic fashion. The horse was walked to distribute the contrast. Following this, a post-contrast scan (T1 3D GRE trans, 5 mm slice thickness) was performed.

### 2.4. Tenoscopic Procedure

All horses subsequently underwent tenoscopy of the DFTS in general anaesthesia. The horses were placed in lateral recumbency with the affected limb positioned uppermost or lowermost, based on the surgeon’s preference, and aseptically prepared for surgery. The tenoscopy of the DFTS was performed using a standard medial or lateral approach by an ECVS board-certified equine surgeon using a 30°-forward-angled, 4 mm rigid arthroscope (Karl Storz GmbH & Co.KG, Tuttlingen, Germany). Briefly, a standard arthroscopic portal was made into the DFTS outpouching between the palmar/plantar annular ligament and proximal digital annular ligament after distention of the DFTS with 40 mL NaCl over the distal pouch. Intraoperatively lesions were classified according to whether an MF lesion was present or not, whether a rupture was partial or complete, and whether it was located medially or laterally.

### 2.5. Image Analysis

Ultrasonographic images and contrast radiographs, as well as the MRI and MRIt, images were randomised within each diagnostic method group. Using a diagnostic workstation and medical imaging software (Intellispace PACS Radiology 4.4553.0, Phillips Healthcare, Zurich, Switzerland), the images were examined by two ECVDI board-certified large animal radiologists. All cases were evaluated on one modality before the evaluation of the next modality in the following sequence: ultrasonography, radiography, MRI, and MRIt. The radiologists were blinded to the findings of the tenoscopy. The radiologists first examined all images separately, and, in cases where they did not agree, a consensus was later reached.

**Figure 1:**
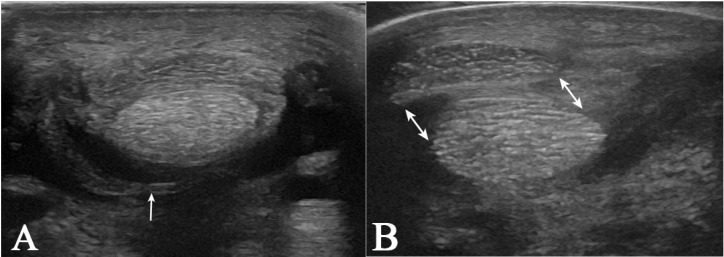
(**A**) Transverse ultrasound image of the fetlock area (medial to the left). There is an asymmetrical thickened and hypoechoic appearance of the lateral aspect of the manica flexoria at its attachment to the superficial digital flexor tendon. The floating edge of the manica flexoria within the synovial fluid of the digital sheath is visible at the lateral aspect (arrow). (**B**) Transverse ultrasound image of the fetlock area obtained on the non-weight bearing limb. There is a medial displacement of the superficial digital flexor tendon relative to the deep digital flexor tendon (arrows).

Ultrasonographically, MF lesions were diagnosed based on previously described imaging features [5,40,41]. Briefly, they included any of the following criteria: incomplete or impossible visualisation of the MF borders; heterogeneous MF echogenicity; irregular MF borders; the presence of loose, retracted, or floating MF edges within the synovial fluid; irregular margins of the superficial digital flexor tendon (SDFT) adjacent to the DDFT; and displacement of the SDFT in a medial or lateral direction relative to the DDFT [5,40,41] (Figure 1).

**Figure 2:**
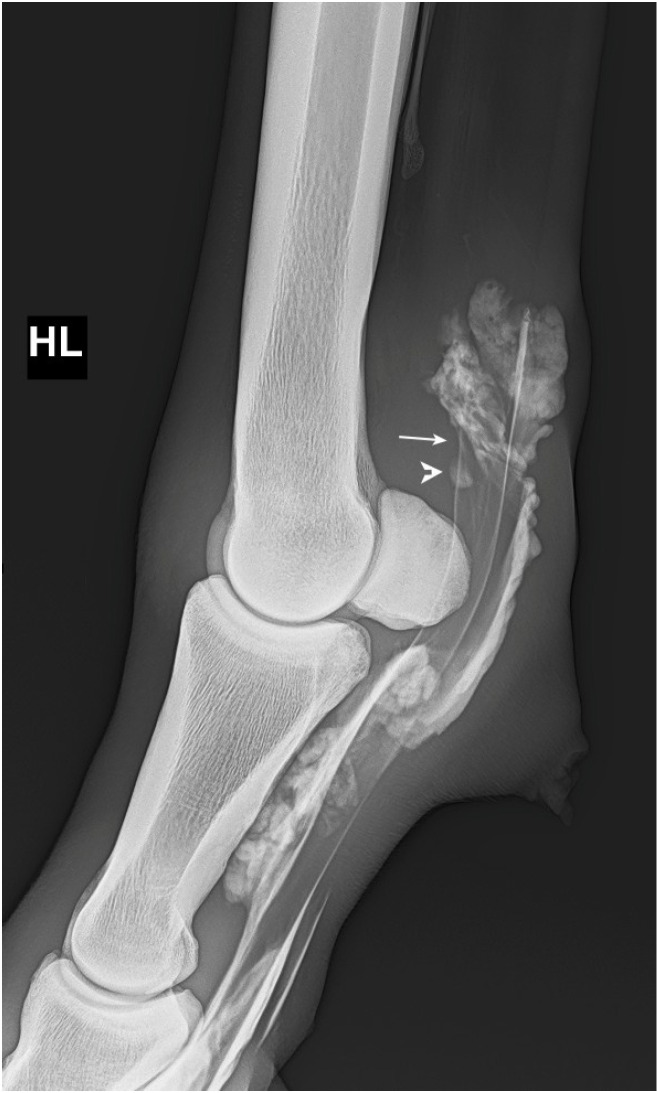
Lateromedial contrast radiograph demonstrating two diagnostic criteria used for assessment of manica flexoria lesions. The two parallel lines which delineate the manica flexoria just above the proximal sesamoid bones, at the dorsal border of the deep digital flexor tendon, are not visible (arrow), and there is an isolated area of contrast overlying the dorsal border of the deep digital flexor tendon at the level of the manica flexoria (arrowhead).

MF lesions were identified via contrast radiography based on the following previously published criteria: loss of the presence of two parallel lines delineating the MF proximal to the proximal sesamoid bones at the dorsal border of the DDFT, loss of the extension of the dorsal line distally to meet or overlie the proximal border of the proximal sesamoid bones, and presence of an isolated area of contrast overlying the dorsal border of the DDFT at the level of the MF [6] (Figure 2).

**Figure 3:**
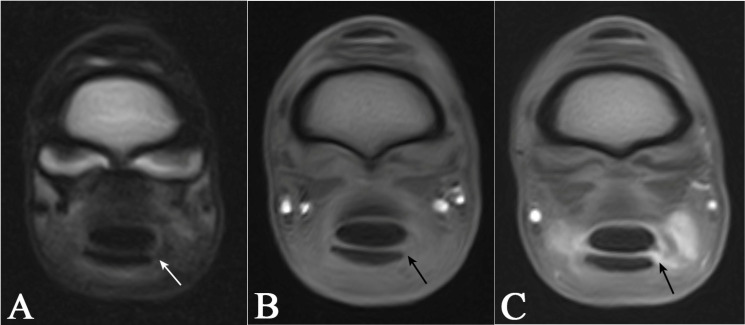
Magnetic resonance images (MRI) indicating a manica flexoria lesion (arrow): transverse T2-fast spin echo sequence (**A**), transverse T1 sequence (**B**), and a transverse T1 sequence following intrathecal gadolinium administration (**C**) of the same limb. In this case, the manica flexoria lesion could only be diagnosed in the transverse post-contrast T1 sequence clearly (**C**). In the T2 sequence, a tear could have been suspected (**A**).

**Figure 4:**
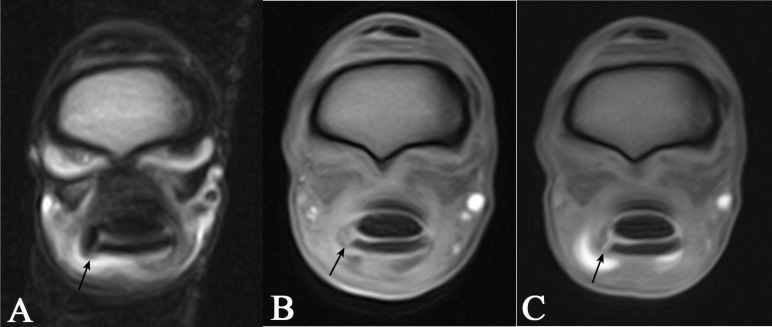
Magnetic resonance images (MRI) indicating a manica flexoria lesion (arrow): transverse T2-fast spin echo sequence (**A**), transverse T1 sequence (**B**), and a transverse T1 sequence following intrathecal gadolinium administration (**C**) of the same limb. The manica flexoria lesion, as well as tendon thickening and rounding, is visible in all sequences.

The MRI/MRIt images were first evaluated solely for diagnostic quality, focusing primarily on artefacts that could compromise image assessment. Further MRIt images were assessed for the amount and distribution of contrast within the DFTS, filling defects, iatrogenic gas artefacts, and contrast extravasation. The reviewer was allowed to alter the window level, width, and zoom. The definition of the normal MRI appearance of the MF was based on the current literature [20,42,43]. A lesion was considered present if synovial fluid or contrast media accumulated within the normal hypointense MF, if there was an exposed attachment point of the MF at the SDFT, or if there were recoiled portions of the MF visible in the DFTS (Figure 3 and Figure 4).

### 2.6. Statistical Analysis

Data were stored in Microsoft Excel. The distribution of data for continuous variables was assessed for normality by use of the Kolmogorov–Smirnov test. Results were reported as the mean ± standard deviation for variables with parametric distribution and median (range) for variables with non-parametric distributions.

Diagnostic performances (sensitivity and specificity) of ultrasonography, contrast radiography, MRI, and MRIt were calculated for MF lesions. Tenoscopic findings served as the reference. Sensitivity and specificity between MRIt and the other modalities were compared using McNemar’s tests. All statistical analyses were performed with a commercially available statistical software programme (v29.0.2.0, SPSS Inc., Chicago, IL, USA), and a *p*-value < 0.05 was considered statistically significant.

## 3. Results

A total of ten horses were included in this study, comprising five mares and five geldings. The breed distribution was as follows: four Warmbloods, two New Forest Ponies, two Spanish breeds, one Icelandic horse, and one Paint Horse. The median age was 14.5 years (range: 6–20 years). Two left forelimbs, six left hindlimbs, and two right hindlimbs were examined. The quality of contrast radiography, ultrasonography, and MRI/MRIt was considered diagnostically sufficient in all horses. Contrast distribution was judged to be very good in both contrast radiography and MRIt. Mild extravasation of contrast media at the injection site was observed in all contrast radiography studies. In six cases, mild flow artefacts were present in the T2-weighted MRI sequences, and in two additional cases, mild motion artefacts were present in the T2-weighted MRI sequences. However, these findings did not impair overall diagnostic interpretation.

Ultrasonography detected four out of seven MF lesions, corresponding to a sensitivity of 57% and a specificity of 33%. Ultrasonography was the only imaging modality in this study that falsely identified an MF lesion in two cases that was not present, resulting in two false-positive results.

Contrast radiography detected five out of seven MF lesions, yielding a sensitivity of 71% and a specificity of 100%. In all five cases, the diagnosis was based on the observation that the parallel contrast lines outlining the MF failed to merge distally. In two of these cases, an additional isolated accumulation of contrast overlying the dorsal border of the DDFT at the level of the MF was observed.

The complete MRI procedure, including sedation, positioning, pre-contrast sequences, contrast administration, and post-contrast imaging, required approximately 45 min per horse.

MRI detected four out of seven MF lesions (sensitivity 57% and specificity 100%). MRIt identified five out of seven MF lesions, demonstrating a sensitivity of 71% and a specificity of 100%. All MF lesions were identified using the previously described criteria. A comparison of imaging modalities using the McNemar’s test revealed no statistically significant differences in the detection rates of MF lesions between MRI and MRIt, MRIt, and contrast radiography, or MRIt and ultrasonography (*p* = 1). Confidence intervals for sensitivity (3.6–96.0%) and specificity (0.84–100%) were wide, reflecting the small sample size.

Tenoscopy was performed in lateral recumbency for all horses, with the laterality selected based on the surgeon’s preference. During tenoscopy, the following lesion prevalences were found: seven MF lesions (one forelimb, six hindlimbs; three medial, two lateral, one distal fibrillation, one central); two DDFT lesions (one forelimb, one hindlimb); and in one horse, a thickened, constricted plantar annular ligament (1 hindlimb) was detected. Six of the MF lesions were complete tears, and, in one, only the distal quarter of the MF was fibrillated.

## 4. Discussion

This study assessed the diagnostic potential of four imaging modalities, standing low-field MRI, MRIt, ultrasonography, and contrast radiography, for the detection of MF lesions in clinical cases, using tenoscopy as the reference. The authors hypothesised that MRIt would most accurately diagnose MF lesions compared to the other imaging modalities. MRIt and contrast radiography both achieved a sensitivity of 71% and a specificity of 100%, each identifying one more MF lesion than a pre-contrast low-field MRI (57.1% sensitivity, 100% specificity) and ultrasonography (57.1% sensitivity, 33% specificity) (Figure 3). Although MRIt demonstrated higher sensitivity than MRI and ultrasound in this study, the differences were not statistically significant, likely due to the small sample size. Therefore, we cannot conclusively state that MRIt outperforms MRI, ultrasonography, or contrast radiography, which showed the same diagnostic performance.

Based on this study, neither MRI nor MRIt improved the detection of MF lesions over established modalities. Both ultrasonography and contrast radiography performed as well or better in terms of sensitivity and specificity. Moreover, contrast radiography and ultrasonography are both performed faster and more cost-effectively than MRI. One of the advantages of having an MRI diagnose an MF lesion is obtaining simultaneous information on the laterality of the lesion pre-operatively. Contrast radiography is not capable of this, but it can be compensated for if ultrasonography and contrast radiography are used in combination.

Nevertheless, cross-sectional imaging modalities like MRI and also CTT definitively have their place in the imaging of MF lesions [11,12,13,20,21,22]. In the author’s clinical experience chronic, longstanding cases of DFTS injuries can be very challenging in terms of pre-operative imaging. They can present with adhesions within the DFTS and substantial synovial membrane proliferation, which both can complicate a clear ultrasonographic diagnosis of an MF tear. Also, thick skin leads to poor-quality ultrasonographic images [7]; for example, robust breeds often need a cross-sectional imaging technique if the laterality of the MF rupture wants to be diagnosed pre-operatively [11]. In many cases, diagnosing the combination of MF ruptures with any other intrathecal tendinopathy, especially marginal tendon lesions, may benefit from a cross-sectional imaging modality.

In this study, MRIt offered no diagnostic benefit over MRI in detecting MF lesions, despite the benefits of a T1 sequence with high spatial resolution and the benefits of the hyperintense synovial fluid due to the contrast. Several anatomical and technical factors likely account for this result. The current study used MRI sequences with a slice thickness of 5 mm and a gap width of 1 mm. This predisposes small MF tears to partial-volume averaging, so only larger, complete lesions are depicted, and then often only just on one or two slices. Also, considering the MF’s average length of 32 mm in the forelimb and 29.4 mm in the hindlimb, this limitation is substantial [44]. The MF consists of a tendinous and an areolar part, and, so far, it is not clear whether the areolar part is detectable in MRI or ultrasonography, as it is very narrow and merges into the DFTS wall. Thus, the length of the detectable tendinous MF, especially on the hindlimb, is further reduced, as the areolar part of the MF in the hindlimb is longer [44]. The MF further tapers from a thick proximal part to a thin distal edge (being <1 mm thick). This distal part has a distally tapering attachment to the SDFT [44]. This part can therefore hardly be differentiated via MRI, even if intrathecal gadolinium enhances the contrast between the tendinous structures and the synovial fluid.

The contrast between different tissue types is dramatically influenced by the MRI sequence selected [24]. As a paramagnetic contrast agent, gadolinium reduces both T1 and T2 relaxation times, with a predominant effect on T1, resulting in brighter signals in enhanced tissues or fluids on fast-acquisition images [25]. Its T1 signal intensity is influenced by both its concentration and the magnetic field strength [26]. While T1-weighted sequences combined with gadolinium are theoretically superior to T2-weighted images as T1-weighted sequences usually have excellent contrast and clear boundaries between different tissues [24,45], it is possible that demonstrating a possible true benefit of MRIt would require a larger cohort to achieve higher statistical power. However, ultimately, the current results are more likely attributed to the inherent limitations of the imaging modality itself, specifically its low spatial resolution and high slice thickness, rather than the sample size of the study.

In a cadaver model, a 3T MRI yielded 85% sensitivity and 96% specificity for the detection of artificially created MF lesions [13]. In contrast, another cadaver study, in which artificially created lesions of the MF were examined in a 0.27T MRI, both with and without gadolinium, reported sensitivities of 61% (MRI) and 50% (MRIt) and specificities of 100% and 96%, respectively [39]. The results of the latter study are comparable and in a similar range to the results of this study. These lower detection rates of low-field MRIs in these two studies likely reflect the thicker slices and increased partial-volume averaging inherent to low-field MRI systems [23,46]. Furthermore, high-field MRI benefits from a greater signal-to-noise ratio and soft tissue contrast, producing better spatial resolution and potentially higher diagnostic accuracy, particularly for small lesions [16,23,47].

Motion artefacts are another critical problem that can significantly affect image quality and, in turn, the diagnostic performance of MRI in standing sedated horses [48]. In the limb, the incidence of motion artefacts increases when examining more proximally located anatomical structures [49]. The MF is located above the PSB and is therefore certainly susceptible to motion artefacts. However, in this study, only two cases showed slight motion artefacts, which did not impair the diagnostic quality of the images obtained. The implementation of motion correction software, such as the iNAV sequences, would likely increase the diagnostic rate of MF lesions in low-field MRI. However, at the time point of the study, these were not available yet, and this remains to be evaluated in the future.

In this study, we diluted 0.5 mL of gadolinium in 20 mL of NaCl to achieve a final concentration of 1.22 mmol/L before injection into the DFTS. This concentration falls within reported ranges: human intra-articular studies most commonly use 2 mmol/L (range 2–5 mmol/L) [33,50,51,52], while optimal levels have been proposed between 0.7 and 3.5 mmol/L [53]. In veterinary research, small-animal models have demonstrated adequate diagnostic performance at even lower gadolinium concentrations [34,54,55].

In case a standing CT machine is available, CT tenography is superior to low-field MRI in diagnosing MF lesions in artificially created lesions. In the aforementioned study, CTT achieved a sensitivity of 85% and a specificity of 96% for the detection of MF lesions [13]. We can assume that CT tenography is likely also better than MRIt, given the lack of significant improvement in the diagnostic performance of MRI when adding intrathecal contrast in this study.

The primary limitations of this study are its small sample size and corresponding low number of MF lesions. Consequently, confidence intervals for sensitivity and specificity were wide, limiting our ability to detect subtle performance differences between low-field MRI, MRIt, and standard imaging modalities. A larger cohort may have revealed statistically significant differences, if present. Also, adding more sequences and planes to the MRI study protocol, instead of using the short protocol as described here, may have achieved a better outcome (higher sensitivity and specificity).

## 5. Conclusions

The MRIt did not enhance the diagnostic performance of low-field MRI to diagnose naturally occurring MF tears in horses. Furthermore, neither MRI nor MRIt demonstrated superiority over contrast tenography, except in the diagnosis of lesion laterality. Compared with ultrasonography, which exhibited a rather low specificity, both MRI and MRIt may better distinguish an intact from a torn MF.

## Data Availability

The original contributions presented in this study are included in the article. Further inquiries can be directed to the corresponding author.
